# Comparison of the accuracy of conventional impression technique and an intraoral scanning system after crown preparation in canine teeth of dogs: a cadaver study

**DOI:** 10.3389/fvets.2025.1683297

**Published:** 2025-10-20

**Authors:** Benjamin Metje, Jerzy Pawel Gawor, Matthias Lüpke, Sebastian Meller, Jan Schreyer

**Affiliations:** ^1^University of Veterinary Medicine Hannover, Foundation, Hannover, Germany; ^2^Tierärztliche Gemeinschaftspraxis am Kaßberg, Dr. Plümer and Dr. Schreyer, Chemnitz, Germany

**Keywords:** intraoral scanner, IOS, digital impression, accuracy, prosthodontics, stone model

## Abstract

**Objective:**

This study aimed to compare digital impressions obtained by an intraoral scanner (IOS) with the dimensions of the corresponding stone model produced from conventional impressions and the original tooth to assess the accuracy of IOS in veterinary dentistry.

**Methods:**

In this cadaver study, 40 canine teeth of dogs underwent standard crown-preparation. For each specimen, both conventional impressions with subsequent stone model fabrication and digital impressions were obtained. The surfaces of the stone replicas and the original teeth were scanned, and the distance between the stone model surface and the original tooth surface, as well as the surface of the intraoral scan and the original tooth surface, was compared. Mean and median surface deviations were computed in millimeters using a 3D surface comparison tool of Amira 3D Pro software (Thermo Fisher Scientific, Waltham, Massachusetts, USA).

**Results:**

Even though the deviations between the stone models and the original teeth were lower compared to the ones between digital impressions and the original teeth in both mean and median, no significant differences in the deviations from the original tooth between both modalities were detected (*p* = 0.4721 for mean values, *p* = 0.4129 for median values).

**Conclusion:**

The observed deviations between digital and conventional impression techniques were minimal and fell within clinically acceptable thresholds as defined in human dentistry literature. These results indicate that this IOS system could provide a reliable and accurate alternative for capturing crown-prepared canine teeth in dogs compared to the currently used standard technique.

## 1 Introduction

For a long time, obtaining conventional dental impressions has been the gold standard for reproducing the oral situation in human dentistry and still is in veterinary dentistry (1–3). The materials and techniques for those impressions got more precise over time. However, particularly in human dentistry, conventional impressions were found to be associated with certain disadvantages (4). For example, impression material such as alginate can cause discomfort or even nausea for patients due to an unpleasant taste, texture or the expansion of material into the pharyngeal region. Errors like minor voids or slight movement during the setting time, prolonged processing time and other factors will alter the results and accuracy (5, 6). Some of these drawbacks do apply in veterinary dentistry (3, 7).

To overcome the limitations of conventional impression techniques, intraoral scanners (IOS) have been developed, employing optical systems within handheld devices to record the surface geometry of oral and dental structures with high spatial resolution, producing detailed three-dimensional models in stereolithography (STL) format. While studies in human dentistry have demonstrated that such digital methods achieve accuracy levels comparable to those of traditional stone models (2, 7–9), their true clinical value becomes particularly evident when considered within the broader context of the digital workflow (2, 8–10). By enabling direct integration with Computer-aided design (CAD) and computer-aided manufacturing (CAM) based laboratory systems, IOS not only reduces chairside time and enhances patient comfort, but also streamlines downstream processes, thus fostering a seamless, efficient, and increasingly standardized collaboration between dentists and laboratory (10).

In veterinary dentistry, treatment and procedures without general anesthesia (GA) are considered to be inadequate and provide a substandard level of care (11). Dental impressions are also required in specific clinical situations, such as the fabrication of full metal crowns following prosthodontic preparation and are therefore taken exclusively under GA. The process of obtaining impressions is technically demanding and holds several potential sources of error (12). The dental laboratory must be provided with impressions of excellent quality to allow for the manufacturing of prosthetic restorations. Even minor inaccuracies can result in a significant misfit of the prosthetic crown that cannot be realized until the next GA. In such cases, patients might often need additional GA as the prosthetic crown cannot be cemented, and the practitioner needs to retake the impression and repeat the fabrication process. That increases both the patient’s anesthetic risk and the financial burden on the owner.

Obtaining digital impressions may eliminate potential sources of error during the conventional impression-taking process, such as voids of material alteration. It also avoids the challenge that arises from the natural, slightly curved shape of the canine teeth in dogs, especially when taking a full arch impression. This shape makes it challenging to obtain an adequate imprint because the impression material must be both elastic and stable enough to follow the curvature without losing the true shape of the canines. In general, digital impressions could streamline workflows, particularly as dental laboratories increasingly adopt digital technologies. The use of IOS-generated STL files could align veterinary prosthodontic procedures with modern laboratory standards. Despite this potential, studies on the applicability and accuracy of intraoral scanning in veterinary dentistry so far are limited to the context of implants and the evaluation of malocclusion (13, 14).

This cadaveric study aims to evaluate the accuracy of an IOS system compared to conventional impressions and stone models (current gold standard) in canine teeth prepared for full-crown restoration using the original tooth surface scanned by a high-resolution desktop scanner as a reference. The authors seek to provide foundational data to support the integration of digital impression systems into clinical veterinary dentistry.

## 2 Materials and methods

For this study, a total of 10 heads of dogs were included. Eight cadavers were obtained from the Department of Small Animal Medicine and Surgery of the University of Veterinary Medicine Hannover, Germany. They were euthanized for reasons unrelated to this study. Two additional dogs used were already deceased upon arrival at the clinic. All cadavers were frozen immediately postmortem/ after arrival at the clinic. Before conducting the study, the heads were separated from the bodies and defrosted for 2 days at room temperature. The study was approved by the local ethics and welfare committee. The owners released the bodies of their pets for scientific purposes to the Clinic for Small Animals at the University of Veterinary Medicine Hannover.

For each head, all four canine teeth (*n* = 40) underwent a reduction of the crown height by cutting the tip of the crown at a random length to simulate a fracture. Subsequently, a crown preparation was performed, creating a chamfer margin using diamond burs (round end taper FG and round 6/8 FG, iM3 dental, Duleek, Ireland) as they would be used in a clinical setting. After finishing the preparation, the teeth were surgically extracted, taking care not to damage the preparation. Within 24 h of extraction, the teeth underwent a disinfection protocol (Peroxy AG+, Stern Weber, Imola, Italy), and after complete drying, one detailed impression per tooth was taken by a resident of the European Veterinary Dental College in his fourth year of training with a medium level of expertise in this field (BM). A hydrophilic addition polymerization type silicon rubber impression material with mixing base & catalyst (iM3 Soft Impression Putty plus iM3 2-part Impression Material, iM3 dental, Duleek, Ireland) was used. Conventional 20 mL syringes were sectioned to the appropriate length corresponding to the prepared crown height, following removal of the plunger. The cut segments were subsequently perforated with multiple openings using a tapered diamond bur (round end taper FG, iM3 Dental, Duleek, Ireland), thereby creating customized yet comparable tray-like devices adapted to each individual tooth.

The two-step impression technique was applied, involving two distinct procedural stages and two different material consistencies/viscosities. In the first stage, a preliminary impression was made using a high-viscosity (putty) silicone material. Equal parts (1:1) of base and catalyst were weighed on a precision scale (3.0 g each material per imprint) and manually mixed, consistent with the manufacturer’s instructions. After setting, a space of approximately 2 mm was created at the preliminary impression of the tooth surface by using sharp instruments. This step is needed to provide room for the second stage, during which a low-viscosity (light-body) material was applied to the putty as well as the clean and dry tooth surface to capture fine details of the preparation. The 2-part Impression Material was dispensed from a cartridge using self-mixing tips provided by the manufacturer. Compared to the monophase technique (using a single viscosity), the two-step method allows for generating higher hydraulic pressure during material insertion, which improves the flow into difficult-to-access areas. This leads to a more accurate reproduction of the clinical situation. All imprints were visually checked for an adequate replication of the preparation and the absence of any defects (BM + trained dental technician). To avoid any influence of transport modality and time, as well as uneven/delayed manufacturing of stone models after impression taking, this process was carried out directly at the dental laboratory.

Within 30 min after complete setting time according to the manufacturer’s instructions, a trained dental technician manufactured conventional stone models of the prepared tooth crowns using hard plaster (Girostone pastel, Amann Girrbach, Mäder, Austria) as well according to the manufacturer’s detailed instructions. The original teeth were then placed into conventional play-dough to mount them in a high-resolution blue-light scanner (ceramill map 600, Amann Girrbach, Mäder, Austria; Supplementary Figure 1). The surface of each tooth was 3D scanned with an accuracy of 0.004 mm to serve as reference geometry (Supplementary Video 1). After the complete setting time according to the manufacturer’s instructions, the stone models were scanned once each, using the same device (Supplementary Figure 2). The crown of each tooth was also scanned with an IOS (Carestream CS 3600, Carestream Dental, Atlanta, USA). The procedure was carried out by an operator familiar with the technique, but without deeper expertise (BM). In cases needed, a thin layer of an anti-reflective spray (Laser Scanning Spray HELLING 3D, Helling GmbH, Heidgraben, Germany) was applied to reduce glare and improve the scanning process. All scans were acquired under the same room ambient conditions to mimic a clinical setting. The intraoral scans were acquired using Carestream CS Imaging Software (Carestream Dental, Atlanta, USA), which automatically identifies regions of insufficient data acquisition and prompts the operator to rescan these areas. It also indicates if the distance between the scanning object and the scanning device would be inadequate to obtain sufficient surface data. Laboratory scans obtained with the Ceramill Map 600 scanner were processed in Ceramill Mind (Amann Girrbach, Mäder, Austria). Both software packages provide real-time feedback on scan quality, allowing targeted rescanning of deficient regions where necessary. This approach ensured that a single, complete dataset was generated for each scanning modality, thereby reflecting conditions comparable to a clinical setting.

All scans were exported as STL-files and imported into Amira 3D Pro software (Thermo Fisher Scientific, Waltham, Massachusetts, USA). To ensure comparability, the area of the pulp opening at the tip of the crown was manually selected and digitally closed using the “fill hole” function in Amira 3D. That created a plane closure in all three scans of each tooth. Also, a rectangular region of interest (ROI) was defined, oriented at the prepared margin of the tooth.

**Figure 1:**
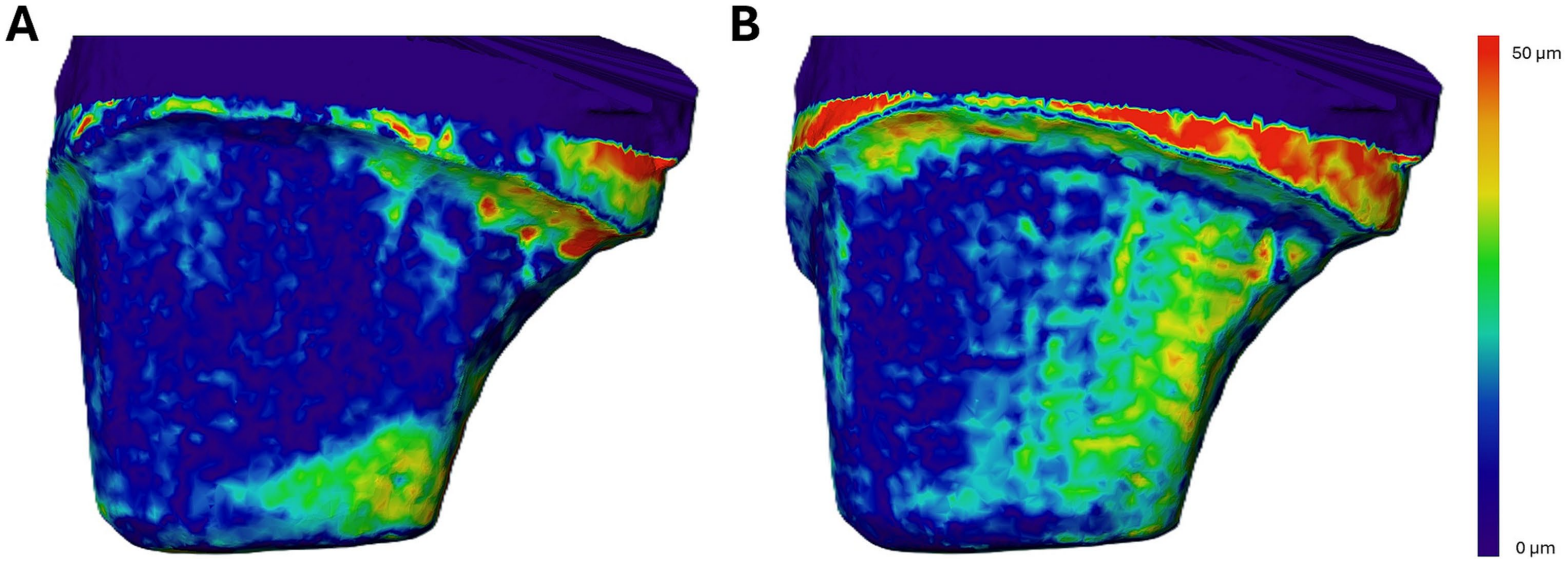
**(A,B)** Comparison of the digital surfaces of an upper crown-prepared canine tooth obtained from two different modalities. The color map represents the distance between each modality’s surface and the reference surface, with values ranging from 0 μm (dark blue) to ≥50 μm (red). Only the labial surface of the prepared tooth is shown. **(A)** Discrepancies between the original tooth surface and the surface of the corresponding plaster model. The highest deviations are observed at the distal preparation margin. **(B)** Distances between the original tooth surface and the surface acquired by the intraoral scanner (IOS). Note that in this case, the largest deviations occur outside the preparation area, which is considered clinically irrelevant.

During the next step, the surfaces of all 3 scans per tooth were aligned. First, a rough manual alignment was carried out, followed by a precise alignment using the “align surfaces” function for the region of interest. A relative root mean square (RMS) threshold of 0.001 was set as the convergence criterion, and the surfaces of the original scan were set as the reference to align to. After successful alignment according to the root mean square value, the “surface distance” function was applied, again designating the scan of the original tooth as the reference surface and using the point-to-nearest-point comparison option. The output of the software included mean and median deviation values in millimeters for every tooth and was calculated for each comparison: original tooth vs. stone model and original tooth vs. IOS (Figure 1).

### 2.1 Statistical analysis

The surface deviation data (mean and median values in millimeters) obtained from each scanning modality—stone model and intraoral scan (IOS) – were organized in Microsoft Excel (Microsoft Corporation, Redmond, WA, USA). To assess the distribution of the data, the Shapiro–Wilk test for normality was performed. As the surface deviation data for both the IOS and stone model comparisons with the original tooth were not normally distributed (*p* < 0.001), non-parametric testing was applied. The Wilcoxon signed-rank test was used to compare paired samples for both groups: (1) original tooth vs. stone model and (2) original tooth vs. IOS. Tests were two-sided, and a significance level of 5% (*p* < 0.05) was applied for all statistical tests.

## 3 Results

The signalment of the cohort included one neutered female, two intact males and two neutered male dogs. The breeds represented were two mixed-breed dogs, one Bearded Collie, one Galgo Español and one German Shepherd. The mean age was 8.85 years (8 years and 10.2 months), ranging from 5 years and 8 months to 17 years and 5 months, while the median age was 6.3 years (6 years and 4 months). All cadavers weighed more than 20 kg (median 26.8 kg, mean 26.68 kg, ranging from 21.0 to 32.4 kg) and were frozen immediately postmortem. In addition, further heads of 5 large dogs of unknown sex, age and breed were included.

### 3.1 Plaster models

**Figure 2:**
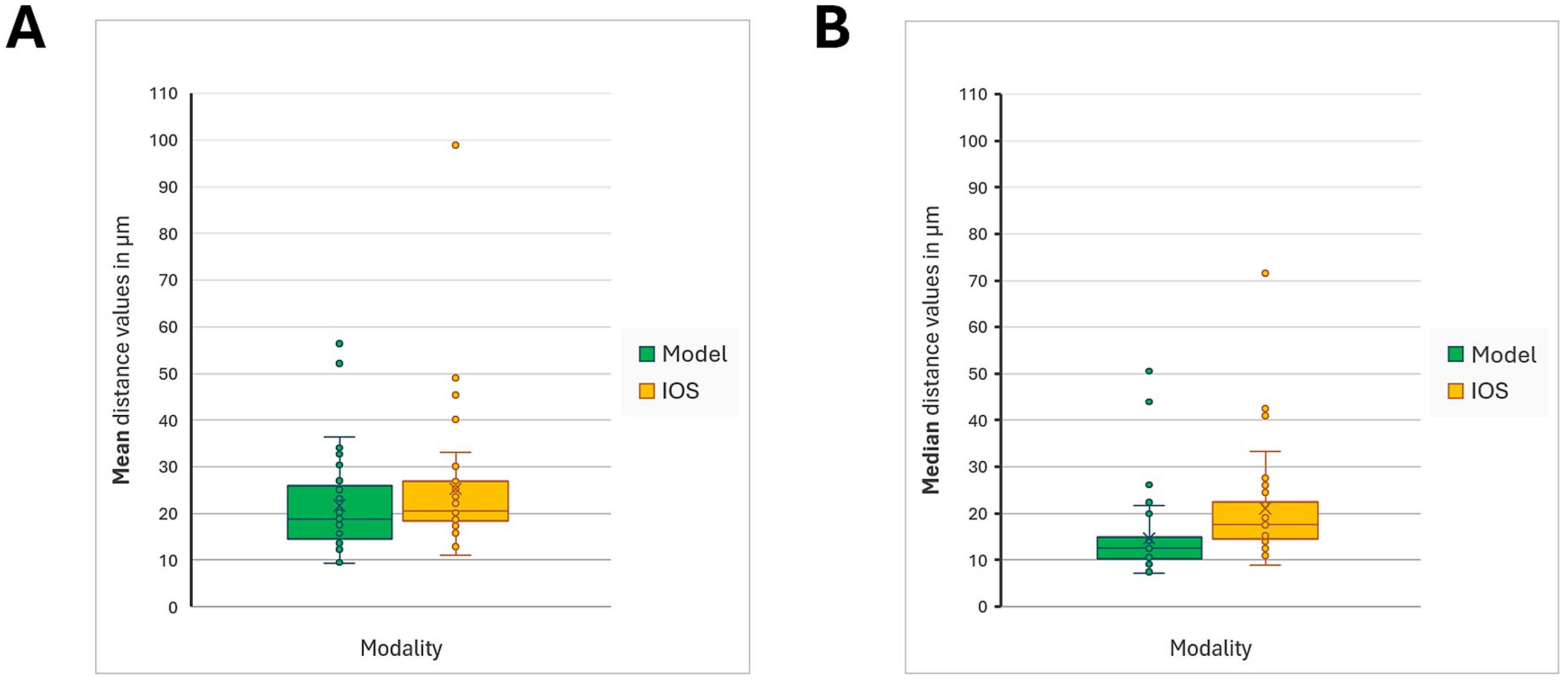
Mean and median distances were calculated for the scans of the original tooth surface and the corresponding imprint modality (conventional stone models / IOS) in 40 crown-prepared canine teeth, using Amira Pro software. **(A)** shows the *mean* distance values for the comparison of the two modalities to the reference surface, while **(B)** shows the *median* distance values. Data distribution was assessed with the Shapiro–Wilk test and, due to non-normality, analysed with the Wilcoxon signed-rank test. No statistically significant differences were found between stone models and intraoral scans for either mean (*p* = 0.4721) or median values (*p* = 0.4129).

The spatial deviations between the scanned surface of the original tooth and the corresponding surface of the plaster model revealed mean values ranging from 9.341 μm to 56.396 μm (Figure 2A). The overall arithmetic mean for this comparison was 21.644 μm, while the median value was calculated at 18.840 μm.

When focusing specifically on the distribution of median values for individual plaster models, the range extended from 7.140 μm to 50.550 μm (Figure 2B). The average of these medians was 14.590 μm, and the median of the distribution was 12.580 μm.

### 3.2 IOS

In the comparison between the original tooth surface and the surface generated by the intraoral scanning (IOS) system, the mean deviation values ranged from 11.049 μm to 90.946 μm (Figure 2A). The calculated overall mean from the dataset (*n* = 40) was 25.230 μm, with a corresponding median value of 20.556 μm.

The analysis of median deviations between the original tooth and IOS surfaces demonstrated a range between 8.950 μm and 71.540 μm (Figure 2B). The mean of these median values was determined to be 21.040 μm, while the median stood at 17.520 μm.

When comparing the mean deviations from the original tooth surface, no statistically significant difference was found between the conventional stone models and the intraoral scanning (IOS) technique. The average deviations did not differ meaningfully between the two modalities (*p* = 0.4721), indicating comparable levels of accuracy across methods concerning mean values.

A similar finding was observed in the analysis of median deviations. The comparison revealed no statistically significant difference between the two techniques (*p* = 0.4129), further supporting the conclusion that both impression methods exhibit equivalent performance in capturing the geometry of the prepared tooth surface.

## 4 Discussion

This study investigated the accuracy with which the Carestream CS 3600 IOS captures the surface geometry of crown-prepared canine teeth and compared the values to the accuracy of traditional impressions and plaster models. Across the 40 samples, the observed differences between the scanned surfaces and the physical reference models—while numerically small—showed no statistically significant variation between the digital and analog techniques. All values for mean and median surface deviations fell well within the clinically accepted margins known from human dentistry, generally cited as under 120 micrometers for marginal gaps and between 20 and 100 micrometers for cement space (15). These results suggest that IOSs may serve as a viable alternative to conventional impression methods in veterinary dentistry, at least in the specific and narrow application of single-crown veterinary prosthodontics.

Although mean surface deviations for the IOS scans (25.230 μm) were slightly higher than those for the plaster models (21.644 μm), this difference was not found to be statistically significant. Both fell below the 30-micrometer mark, a range considered high-fidelity in three-dimensional surface reproduction (15, 16). While the study did not break accuracy down into trueness (systematic error) and precision (random error), its approach aligns with prior findings in human studies, which consistently demonstrate comparable accuracy between digital and analog impressions in different settings. From this, we can cautiously extrapolate a comparable potential in veterinary settings (8–10, 13).

One of the main advantages of digital methods is their capacity to reduce the number of error-prone steps inherent in traditional workflows of taking impressions (17). Errors may occur even more often in impressions taken by veterinarians, as their experience is mostly lower compared to human dentists due to a lower caseload, which indicates dental impressions. Different studies showed the impact of experience on the outcome of conventional impression methods (1, 2, 12). Despite the experience of the person who takes the impressions, conventional impressions involve multiple material transformations: from putty to plaster and finally to a scanned surface, which is necessary in modern laboratories to apply the CAD/CAM technology in the process of prosthetic crowns. Each transformation, including the disinfection process and others, introduces potential distortions (3, 5–7). Digital impressions streamline the process into fewer steps, cutting down on cumulative inaccuracies. In veterinary contexts, where prosthodontic procedures are less frequently performed than in human dentistry, simplifying workflows may be especially helpful. Veterinarians with limited experience in dental impressions, as an impact factor on the outcome of accuracy in dental impressions, might benefit from the more forgiving and guided feedback that digital systems offer during scanning. Some studies showed that the experience of the operator has an impact on the factor “time,” but not on the quality of the achieved scans (18). Other studies from the human field showed contrary findings and highlighted the impact of the operator’s experience and skills on the accuracy of digital impressions (19, 20). Studies in veterinary medicine on these aspects are missing and would be a valuable addition for the use of those devices in our profession.

Digital impression techniques also offer logistical advantages. STL files generated by IOSs can be immediately transferred to dental laboratories, eliminating the risk of distortion or damage during physical transport. The growing integration of CAD/CAM systems in laboratories means this direct compatibility can reduce turnaround time and improve coordination between the veterinary clinician and the dental laboratory, and it has been shown to be a good option in modern veterinary dentistry (21). Real-time visual feedback during scanning also adds diagnostic advantage as clinicians can identify missing data and correct it during the initial anesthesia, avoiding the need for repeated impressions in case of an insufficient outcome of conventional impressions. This would cause a need for an additional anesthesia session, which is costly, stressful and comes with extra risks for veterinary patients.

Despite these highlighted benefits, the limitations of this study need to be taken into account. All scans were conducted using only one IOS model (Carestream CS 3600). Though previous literature in the human field showed a good accuracy for the used model, there are studies suggesting that not all IOS devices on the market perform equally, though in a comparable range of accuracy (22–24). Differences in scanning technologies across manufacturers seem to affect the performance of the different devices (25). Another point to consider is the use of sprays to reduce surface glare that introduces further variables to this study. Their application can differ depending on user technique, affecting thickness and distribution, which may subtly influence results. While some studies suggest scanning sprays can improve accuracy when applied consistently, their variable nature remains a concern for reproducibility (26). In veterinary dentistry, particularly in smaller patients, the restricted intraoral space might represent a relevant limitation. Since intraoral scanner (IOS) tips must maintain a certain size to enable the scanning technique, the dimensions of the tip may hinder adequate access. This constraint is especially pronounced in the caudal regions of the oral cavity, such as the fourth premolar or the first molar of small-breed patients.

Another aspect that must be taken into account is the ex vivo nature of this research. The cadaveric heads, while useful for controlling conditions, do not replicate challenges that have been shown to influence the accuracy in humans, such as salivation, blood, movement, and the variable visibility and access in the oral cavity (27). These real-world factors could compromise scanner performance in our patients. As such, the current data are promising but preliminary, pending *in vivo* confirmation through further research.

This study focused exclusively on single-tooth preparations. While this is a clinically relevant indication, as crowns are often applied individually, it’s important to note that scan accuracy tends to decline as the scanned area increases (3). Human studies have shown that errors accumulate with longer scans, especially when capturing full arches. Canine dental arches are typically larger and more anatomically varying than human ones, so similar challenges may apply or even might intensify in veterinary settings (9, 19, 24). Hence, applying these results to multi-tooth or full-arch scenarios would be speculative at this stage.

From a methodological standpoint, the use of mean and median deviation values offers a broad perspective on scan accuracy. A more nuanced analysis separating trueness and precision would allow future studies to isolate whether deviations arise from consistent bias or unpredictable fluctuation. This distinction might be a matter clinically, particularly in the fit and longevity of restorations (24, 28).

Cost and accessibility also warrant discussion. Intraoral scanning systems represent a significant initial investment in hardware and training. While these costs may be balanced over time through efficiency gains, less need for repeat impressions, faster workflows, and fewer consumables, they could deter adoption by veterinary practices. Group practice models or third-party scanning services might help reduce the economic barrier.

Overall, our data indicate that intraoral scanning can match the accuracy of conventional methods for single-crown restorations in dogs, under controlled conditions. When used correctly, the digital method appears to offer not only equivalent accuracy but also adds workflow advantages. For veterinary dentistry, still in the early stages of digital adoption, these benefits could be transformative, particularly in improving standardization and reducing reliance on operator technique. Future research should focus on expanding the clinical scope: trials involving live animals, full arches, and diverse anatomical situations will better clarify when and where IOS systems are most useful. Comparing different scanner models under identical conditions would also be valuable, as would outcome-based studies tracking the success of restorations produced via digital impressions. A first step could also be the evaluation of the accuracy in a more than single-tooth application and/or the application on premolar and molar teeth of dogs.

In conclusion, while this study provides important evidence supporting the clinical validity of IOS technology in veterinary single-tooth applications (canine teeth), it should be viewed as a foundational step. Broader investigations are needed to solidify its place in routine clinical practice. Yet the path forward is clear: the integration of digital dentistry into veterinary workflows holds promise, and with appropriate research, training, and technological refinement, it could soon become a reasonable addition to the dental care of our veterinary patients.

## Data Availability

The raw data supporting the conclusions of this article will be made available by the authors, without undue reservation.

## Supplementary material

SUPPLEMENTARY FIGURE 1 Original tooth specimen 7/204, which was stabilized in playdough on the rotating platform of the ceramill map 600 during the scanning procedure.

SUPPLEMENTARY FIGURE 2 Illustration of the stone model of specimen 7/204, mounted on the rotating platform of the ceramill map 600 for high-precision surface scanning.

SUPPLEMENTARY VIDEO 1 Demonstration of the scanning procedure of the original tooth specimen 7/204 using blue light grating technology of ceramill map 600, capturing the surface with an accuracy of 0.004 mm.
