# Supplementary figures and images for: Comparison of the accuracy of conventional impression technique and an intraoral scanning system after crown preparation in canine teeth of dogs: a cadaver study
Source: Front Vet Sci. 2025 Oct 20;12:1683297. doi: 10.3389/fvets.2025.1683297 (PMC12580654; doi:10.3389/fvets.2025.1683297)

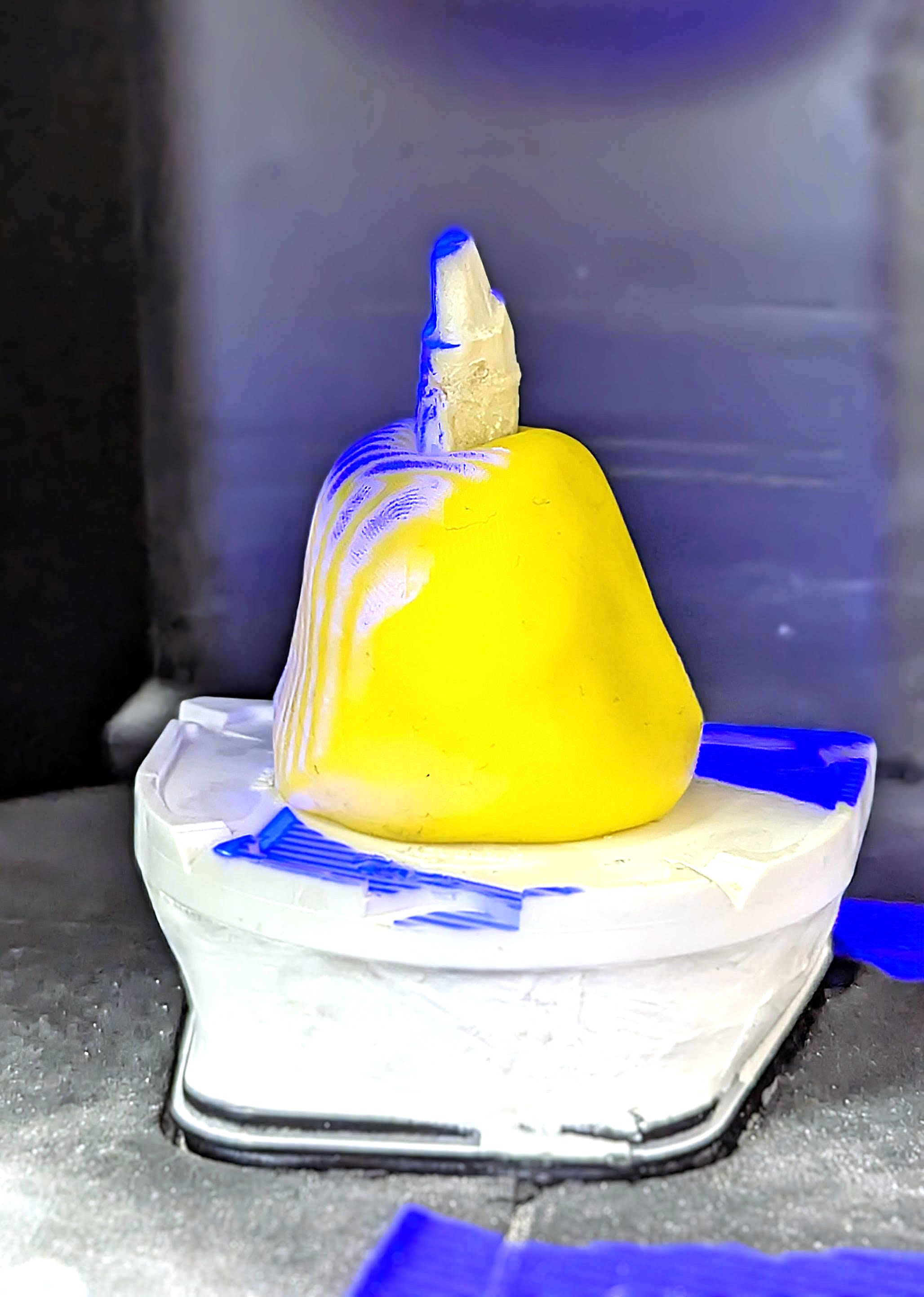

Supplement: Supplementary file 1 [file Image_1.jpeg]

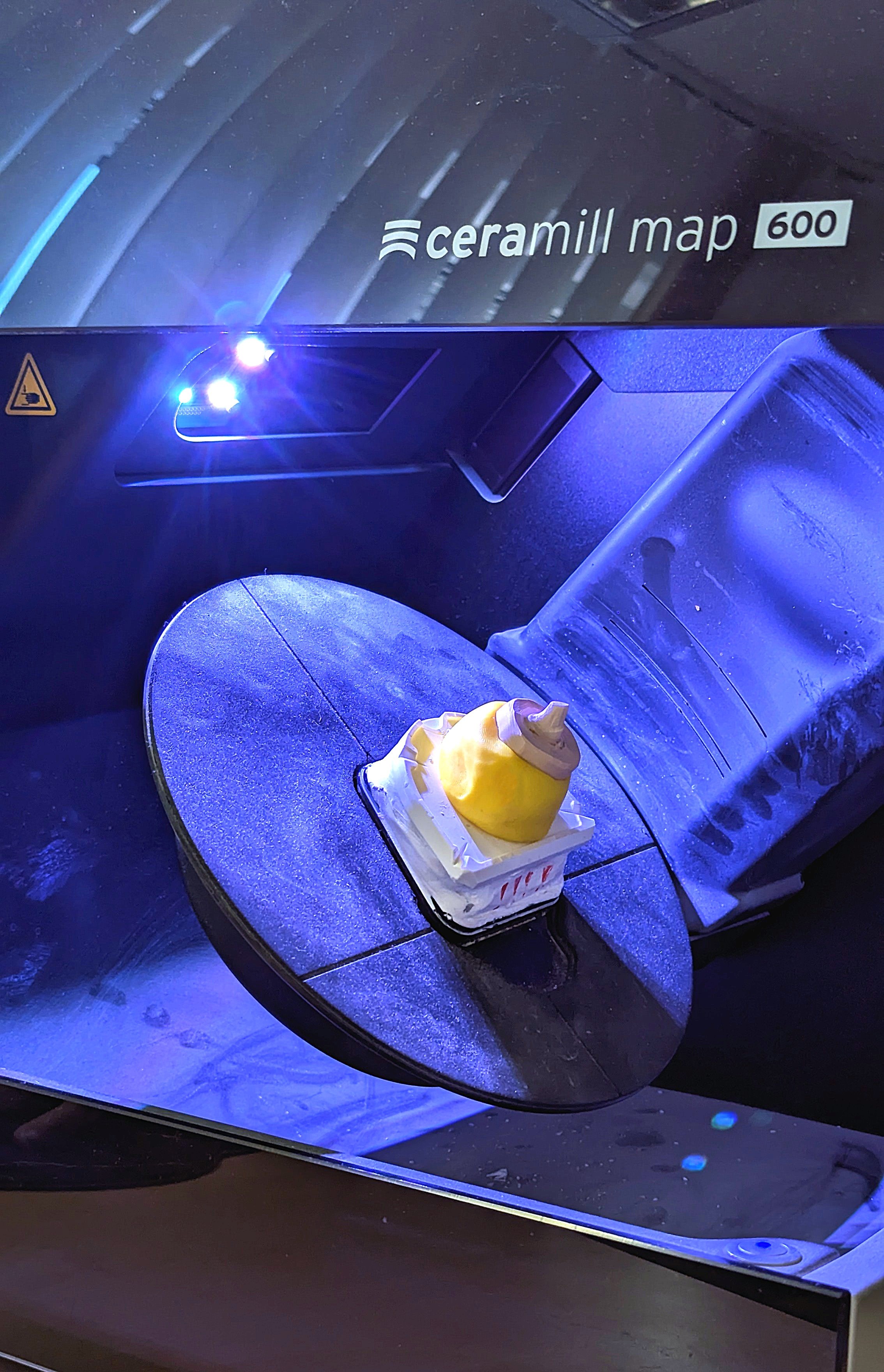

Supplement: Supplementary file 2 [file Image_2.jpeg]
